# Comparative study of Saudi and Turkish coffee consumption in Saudi Arabia: insights into sociodemographic factors and caffeine intake

**DOI:** 10.3389/fnut.2024.1407590

**Published:** 2024-10-09

**Authors:** Nahla Mohammed Bawazeer, Faisal Binsunaid, Atheer Alraqea, Mazen M. Al Fayez, Omar Alhumaidan, Ghadir Fallata, Rehab Aldahash, Nada Benajiba

**Affiliations:** ^1^Department of Health Sciences, College of Health and Rehabilitation Sciences, Princess Nourah Bint Abdulrahman University, Riyadh, Saudi Arabia; ^2^Saudi Food and Drug Authority, Riyadh, Saudi Arabia; ^3^Unité Mixte de Recherche en Nutrition et Alimentation URAC 39 (Université Ibn Tofaïl–CNESTEN), RDC-Nutrition, Kénitra, Morocco

**Keywords:** caffeine consumption, adult, Saudi Arabia, Saudi coffee, Turkish coffee

## Abstract

**Background:**

Saudi and Turkish coffee consumption in Saudi Arabia is increasing considerably, and the nationwide consumption patterns need elucidation to determine the contributions of Saudi and Turkish coffee toward nutrition and health.

**Aim:**

To describe the frequency and quantity of Saudi and Turkish coffee consumption and assess their association with sociodemographic factors.

**Methods:**

This cross-sectional study included 1,030 participants (Saudi Arabia) recruited via an online questionnaire study that collected information on general characteristics and coffee consumption. The caffeine content in Saudi and Turkish coffees was quantified using a standard laboratory technique. The total caffeine intake and exposure were calculated. Associations between the different parameters were assessed.

**Results:**

Significant differences were observed in several demographic and sociodemographic factors according to the frequency of coffee intake. Specifically, individuals who consumed Saudi coffee almost every day (approximately 40% of respondents) were more likely to differ in age, body mass index, marital status, work status, monthly income, and region compared to those who consumed it less frequently. Additionally, one-third of the respondents consumed Turkish coffee, and the frequency of its consumption showed significant differences according to age, nationality, marital status, educational level, and region. Notably, the highest caffeine exposures were 0.95 mg/kg/d for Saudi coffee, 1.31 mg/kg/d for Turkish coffee, and 2.07 mg/kg/d for both coffees combined. The mean contribution to the 400 mg daily caffeine intake limit was significantly higher for Saudi coffee compared to Turkish coffee (*p* < 0.05).

**Conclusion:**

Saudi and Turkish coffee consumption patterns vary across sociodemographic characteristics, where Saudi coffee is generally more consumed. Our study may form a basis for nutrient education in terms of coffee consumption to promote a healthy lifestyle.

## 1 Introduction

After water, coffee is the most frequently consumed beverage worldwide, with more than 2.25 billion cups of coffee consumed daily, and this is reflected by the annual income of the coffee sector estimated to exceed $200 billion (1). Coffee is the second most exchanged commodity worldwide, after oil (2). Coffee contains high levels of antioxidant and anti-inflammatory molecules and is a major source of caffeine (3, 4), a molecule that constitutes nearly 2% of coffee. Caffeine exerts an antagonistic action on adenosine receptors in the brain, leading to central nervous system stimulation (5) and is one of the most comprehensively studied ingredients in food products. Caffeine consumption is associated with improved memory, mood, and cognitive performance (6–9). In addition to physiological studies, epidemiological studies have suggested that caffeine consumption might benefit human health, and a noticeable reduction of risk of chronic diseases, such as type 2 diabetes mellitus, Parkinson’s disease, and liver disease, has been observed among habitual coffee consumers (10, 11).

The global coffee consumption is increasing progressively, including in Asia (12). For instance, Butt and Sultan (2) reported that the average coffee consumption in the Kingdom of Saudi Arabia is 1.6 kg *per capita* per year (2). Saudi Arabia has been experiencing a considerable increase in caffeine consumption. Official statistics revealed that, in 2015, 18,000 tons of coffee were imported to Saudi Arabia, amounting to a total cost of USD 54 million (13). In 2020, the Observatory of Economic Complexity revealed that Saudi Arabia ranked 18^th^ among the countries that were importing the largest amounts of coffee worldwide (14). This increase was further promoted by a couple of governmental initiatives: “The Year of Saudi Coffee” in 2020 (15) and the launch of the Saudi Coffee Company, which was supported by the Saudi Public Investment Fund through a 1.2 billion Saudi Arabian Riyal (SAR) investment (16). Coffee consumption has substantial social importance, and the types of coffee used vary widely across countries (17); Arabic coffee (*Coffea Arabica* or *Rubiaceae*) is one of the most consumed hot beverages in Saudi Arabia, as it is integrated within Saudi tradition and dietary habits. Recently, the name of this coffee has been officially changed to Saudi coffee in Saudi Arabia (18). Butt and Sultan (2) suggested that a typical adult may consume 60–300 mL of coffee in one sitting. Besides Saudi coffee, Turkish-style coffee (prepared in a cezve using very finely ground coffee beans without filtering), which is consumed in countries such as Turkey and the Middle East, is also consumed in Saudi Arabia. Turkish coffee imports have increased from approximately 10 tons annually in 2014 to 50 tons annually in 2016 (19). A study conducted in Jeddah reported that almost half the participants drank Turkish coffee five times or more per week (20). According to the US Food and Drug Administration, the recommended maximum daily intake of caffeine among adults is 400 mg/day (21).

Despite some research on coffee consumption in Saudi Arabia (22–26), a comprehensive evaluation of the frequency and quantity of Saudi and Turkish coffee consumption in association with sociodemographic characteristics across the five Saudi Arabian regions has not been conducted yet, and even less is known about a comparison of these patterns between Saudi and Turkish coffees. Furthermore, an estimation of caffeine consumption based on laboratory test quantification and detailed analysis of consumption has not been reported so far. As the coffee industry in Saudi Arabia continues to grow, there is a concern about the potential increase in caffeine consumption. Therefore, it is crucial to determine the contribution of Saudi and Turkish coffee to the recommended caffeine limit of 400 mg/day.

The primary objective of this study is to describe the main patterns (frequency and quantity) of coffee consumption in Saudi Arabia. The secondary objectives include assessing the relationship between coffee consumption and socio-demographic factors such as body mass index (BMI), marital status, educational level, monthly income, employment status, and age groups. Additionally, the study aims to determine the caffeine consumption per adult and whether it aligns with the recommended guidelines. Our study might help rationalise coffee consumption as part of nutrient education to promote a healthy lifestyle.

## 2 Materials and methods

### 2.1 Study design and study population

This cross-sectional online questionnaire-based study was conducted in Saudi Arabia from September 2021 to January 2022 using the snowballing technique for recruitment. The survey was distributed through social media platforms. The inclusion criteria for the study were being a Saudi citizen or resident in Saudi Arabia aged 15 years or older. The exclusion criterion was the presence of any condition that might affect coffee consumption, such as pregnancy or osteoporosis. Moreover, teenagers aged 15–18 years were included due to previous studies (27, 28) confirming a high rate of coffee consumption in this age group. Participants gave their consent by pressing the “I agree” button before starting the survey. Participation was anonymous and voluntary, and participants could withdraw at any time while responding to the questionnaire. The study information collected from the participants was kept strictly confidential in password-protected files on secure servers, and no information was disclosed to any party without explicit written consent from the principal investigator. This study was approved (IRB no. 21-0288) by the Ethics Research Committee of Princess Nourah Bint Abdulrahman University (Riyadh, Saudi Arabia).

### 2.2 Sample size calculation and sampling technique

A convenience sampling method was initially used by distributing the survey link to participants who were readily accessible. Subsequently, a snowball sampling technique was employed, with these initial participants further disseminating the survey link to others within their network, thereby expanding our sample size. The sample size calculation was performed using the following equation for the total population:


$$\left(n\right)=Z^{2}\;pq/d^{2}$$

where the total population comprised Saudi adults (*n* = 25,828,206; ≥15 years old), with a 95% confidence level *z*-score of 2.576; *p* was the prevalence of the factor under study that was estimated to be 50%; *q* = 1 − *p* = 50%; and *d* was the margin of error = 5%. The minimum number of participants required for the study was 1,083, which included both Saudi citizens and non-Saudi residents, aged ≥15 years. Participants were asked to complete a publicly distributed electronic questionnaire that was shared via various social media applications, such as Twitter and WhatsApp.

### 2.3 Study instruments

The questionnaire included the following sections:

#### 2.3.1 Demographic characteristics

This section aimed to measure general characteristics, such as age (years), sex (male or female), nationality (Saudi or non-Saudi), marital status (unmarried [single, divorced, or widowed] or married), education level (lower than high school, high school, bachelor’s degree, or higher than bachelor degree), employment status (student, working [employed, self-employed], or not-working [not-working, retired, or housewife]), monthly income (less than 2,000, 2,000–5,000, 5,000–7,000, 7,000–10,000, or more than 10,000 SAR), Saudi region (Central region, Northern region, Southern region, Western region, or Eastern region), and health status (with no previously diagnosed disease, or with any listed chronic disease such as anemia, diabetes, hypertension, stomach ulcer, osteoporosis, etc., and included an open-ended option for other diseases). In addition, height and weight were self-reported by the participants, and Body Mass Index (BMI) was calculated and categorized to define weight status as follows: underweight (BMI < 18.5 kg/m^2^), normal weight (BMI 18.5–24.9 kg/m^2^), overweight (BMI 25–29.9 kg/m^2^), and Obese (BMI ≥ 30 kg/m^2^).

#### 2.3.2 The food frequency questionnaire (FFQ)

The FFQ was adopted from the 2010 Caffeine Consumption Questionnaire (CCQ) of the University of North Carolina Wilmington (29) to collect caffeine consumption data, which were modified according to Saudi consumption. This section consisted of questions related to the frequency and quantity of each drink consumed. In terms of frequency, the options were four categories per day (1, 2–3, 4–5, and > 6 times), three categories per week (1, 2–4, and 5–6 times), two categories per month (<1 time and 1–3 times), and never consumed. The suggested options for the quantity consumed were 350, 200, 100, 75, or 60 mL. To unify the quantity and make it easy for respondents to choose the appropriate answer, the questionnaire was supported by photos of different volumes for consumption. For review and accuracy, the questionnaire was shared with five independent experts with PhDs and MSc in Nutrition and Dietetics. Furthermore, a pilot study was conducted to empirically test the questionnaire with 50 participants to ensure the clarity of the questions, refine the food list, and modify the questionnaire. The main changes included reorganizing the frequency question for Saudi/Turkish coffee to prevent confusion and replacing “less than once a month or I do not consume” with “I do not consume” to simplify responses and scoring. Finally, the type of coffee was explicitly mentioned in every question regarding frequency and quantity to ensure accurate responses.

### 2.4 Laboratory analysis for caffeine content in Saudi and Turkish coffees

#### 2.4.1 Chemicals

The chemicals used to quantity the caffeine in the preparations of Saudi and Turkish coffees were ascertained as follows.

Caffeine, as a reference standard (purity 99%), was obtained from Chem Service (West Chester, United States); 99% high-performance liquid chromatography (HPLC)-grade acetonitrile, methanol (purity <99.9%) assay, and glacial acetic acid (purity <99.8%) was obtained from Merck (Darmstadt, Germany); ammonium acetate (purity <98%) was obtained from Panreac AppliChem (Darmstadt, Germany); and magnesium oxide (purity <99.99%) from Sigma Aldrich (Darmstad, Germany). Ultrapure water was obtained from a Millipore system (AVIDITY SCIENCE-Womal Park, UK) in the laboratory.

#### 2.4.2 Equipment

High-performance liquid chromatography with diode array detection (HPLC-DAD) (Agilent Technologies, Santa Clara, CA, United States) was used for the analysis of caffeine. For the analytical separation, we used Zorbax Eclips XDB-C18 (4.6 × 150 mm, 3.5 *μ*, Agilent Technologies) and guard column Eclips XDB-C18 (4.6 × 12.5 mm, 5 μ, Agilent Technologies) at 30°C. Chromatographic separation was performed on a 28-min run at a flow rate of 0.700 mL/min for 28 min. Gradient Mobile Phase pumping was used (90% of mobile phase A [MPA] at 0 min, 90% of MPA at 5 min, 80% of MPA at 7 min, 50% of MPA at 14 min, and 40% of MPA at 18 min, 15% of MPA at 19 min, 15% of MPA at 23 min, 90% of MPA at 24 min, and 90% of MPA at 28 min). The signal was detected at 210 nm (30).

#### 2.4.3 Preparation of standard and reagent formulations

Considering the purity of caffeine, stock standards (1,000 mg/L) were prepared (3:1:1) in Class A filled with Extraction Solution (ES) from deionised water, methanol, and acetonitrile. Two sets of calibration standards were prepared, with concentrations of 0.5–10 mg/L for the first set, and concentrations of 25–100 mg/L for the second set. The MPA (10 mM ammonium acetate and 0.05 acetic acid) was prepared by dissolving 0.77 ± 0.002 g ammonium acetate in 800 mL deionised water, followed by the addition of 500 μL acetic acid to the solution to obtain a volume of 1 L (durable for 7 days). Mobile phase B comprised 0.1% acetic acid in methanol. The mobile phase was degassed for 12–15 min in an ultrasonic bath (Elma, Germany). An ES was prepared (deionised water:methanol:acetonitrile, 3:1:1).

#### 2.4.4 Sample preparation and caffeine content analysis of Saudi and Turkish coffees

The liquid sample was diluted (1:10) with ES. The mixture was vortexed using a vortex mixer (Velp, Canada) for 1 min and sonicated for 10 min. Then, the supernatant was filtered using a Nylon 0.45-μm syringe filter (VWR, United States), and the filtrate was transferred into a 2 mL HPLC vial from which 6 μL was injected into the HPLC system.

Owing to differences in content based on different types and degrees of roasting, three different types of Saudi coffee (Khawlani Yemeni, Khawlani, and Harari) and three different types of Turkish coffee with different degrees of roasting (light, medium, and dark) were used for analysis. The standardised preparation method involved boiling 20 g coffee beans in 500 mL water for 10 min.

After grinding approximately 30 g of coffee beans, samples of 0.05 and 2 g were accurately weighed. Magnesium oxide mixed with 200 mL hot water (approximately 90°C), was stirred with a magnetic stirrer (NUOVA, United States) for 20 min at low speed and covered during stirring by a parafilm (Bemis, United States), and then left to cool. The solution was filtered using a Nylon 0.45-μm syringe filter (VWR, United States), and the filtrate was transferred into a 2 mL HPLC vial from which 6 μL was injected into the HPLC system. The reference values of Saudi and Turkish coffee caffeine content were obtained from the SFDA laboratory and were confirmed as references after ensuring the availability of collection, preparation, and analysis of information for each product. The mean caffeine content was 49.8 mg/100 mL for Saudi coffee and 129.6 mg/100 mL for Turkish coffee.

### 2.5 Statistical analysis

Data were entered into the computer and analysed using IBM SPSS software package version 20.0 (IBM Corp, Armonk, NY, United States). Qualitative data are presented as numbers and percentages. The Kolmogorov–Smirnov test was used to verify the normality of the distribution. Quantitative data were described using the mean and standard deviation. To measure the amount of caffeine exposure, the total caffeine intake was calculated based on the reported frequency and amount of coffee consumption by using the formula (31):


$$\begin{array}{l} \mathrm{Dietary exposure}\\ =\frac{\sum \mathrm{Concentration of chemical in food}\times \mathrm{Food consumption}}{\mathrm{Body weight}\;\left(\mathrm{kg}\right)} \end{array}$$

The results were calculated as a percentage of the recommended caffeine consumption limit per day (400 mg). The chi-square test was used to investigate the associations between categorical variables. Monte Carlo correction is performed for the chi-square results when more than 20% of the cells have an expected count of less than 5. The Mann–Whitney *U* test was applied for non-normally distributed quantitative variables to compare the two study groups, whereas the Kruskal–Wallis *H* test was applied for non-normally distributed quantitative variables to compare more than two studied groups. Finally, the Wilcoxon signed-rank test was performed for non-normally distributed quantitative variables to compare Saudi and Turkish coffee consumption among study participants. The results obtained were considered significant at a *p*-value ≤0.05.

## 3 Results

### 3.1 Sociodemographic characteristics

A total of 1,037 respondents completed the questionnaire, of which, 1,030 met the inclusion criteria and provided all the answers. Table 1 summarises the sociodemographic characteristics of the study population (*n* = 1,030). More than two-thirds (69.3%) of the participants were female. The mean age was 35.63 ± 12.61 years with the two age groups, 20–29 and 30–39 years, predominantly represented 36.2 and 24.2% of the study sample, respectively. In terms of BMI, approximately 40% were normal and 30.7% were overweight. Obese participants represented almost one-fourth of the study population. The majority of participants were Saudis (95.7%), slightly more than half (51.6%) were married, and this proportion was similar to that of the working status (52.2%); 61.2% of respondents had a bachelor’s degree, and 38.2% of participants had a monthly income of more than 10,000 SAR. Respondents from the central region comprised the largest proportion of the study population (61.8%). Regarding the health status, nearly three-quarters of the participants (74.1%) self-reported being healthy (i.e., not diagnosed with any particular disease).

**Table 1:** Sociodemographic characteristics (*n* = 1,030).

Sociodemographic data	No.	%
Gender		
Male	316	30.7
Female	714	69.3
Age (years)		
15–19	39	3.8
20–29	373	36.2
30–39	256	24.9
40–49	184	17.9
50–59	130	12.6
≥60	48	4.7
BMI (kg/m^2^)		
Underweight	59	5.7
Normal	407	39.5
Overweight	316	30.7
Obese	248	24.1
Nationality		
Saudi	986	95.7
Non-Saudi	44	4.3
Marital status		
Unmarried (single, divorced, or widowed)	499	48.4
Married	531	51.6
Educational level		
Lower than high school	35	3.4
High school	151	14.7
Bachelor’s degree	630	61.2
Higher than a bachelor’s degree	214	20.8
Work status		
Student	197	19.1
Working (employee + self-employed)	538	52.2
Not working (not working + retired + housewife)	295	28.6
Monthly income (SAR)		
<2,000	275	26.7
2,000–5,000	159	15.4
5,000–7,000	75	7.3
7,000–10,000	128	12.4
>10,000	393	38.2
Region		
Central	637	61.8
Northern	36	3.5
Southern	44	4.3
Western	213	20.7
Eastern	100	9.7
Health status		
Healthy (no disease)	763	74.1
Unhealthy (diseased state)	267	25.9

BMI, Body mass index.

### 3.2 Frequency of Saudi coffee consumption

A total of 902 participants (88%) confirmed that they drank Saudi coffee, regardless of the frequency of consumption. Approximately half of the respondents confirmed that they consumed coffee every day. Additionally, the most common serving size of Saudi coffee consumed was 60 mL (49%) (Table 2). Table 3 shows the frequency of Saudi coffee consumption by sociodemographic factors. There were significant differences in age, BMI, marital status, work status, monthly income, and region according to the frequency of Saudi coffee consumption (<0.05). The age group 20–29 years showed the highest proportion of never consuming Saudi coffee (42.2%). The highest frequency was observed within a frequency of 1 time per day among the groups aged 40–49 and 50–59 years (25.2 and 18.4%, respectively). The highest frequency of Saudi coffee consumption, ≥6 times per day, was observed in normal-weight participants (55.3%), unmarried participants (73.7%), students (42.1%), and those with the lowest monthly income (<2,000 SAR) (39.5%). According to the marital status, among the married participants, 63.4% of consumers reported intake of Saudi coffee 2–3 times a day, and 48.8% of these had an income >10,000. The respondents from the central region showed the highest percentage of frequency of Saudi coffee consumption (≥6 times per day at 60.5% and 1 time per day at 71.8%).

**Table 2:** Distribution of the studied cases according to frequency and quantity in Arabic coffee and Turkish coffee (*n* = 1,030).

	Saudi coffee	Turkish coffee
	No. (%)	No. (%)
Frequency		
≥6 Times/days	38 (3.7)	0 (0.0)
4–5 Times/days	43 (4.2)	5 (0.5)
2–3 Times/days	205 (19.9)	23 (2.2)
1 Time/day	206 (20.0)	43 (4.2)
5–6 Times/weeks	35 (3.4)	13 (1.3)
2–4 Times/weeks	160 (15.5)	34 (3.3)
1 Time/week	115 (11.2)	53 (5.1)
1–3 Times/months	100 (9.7)	170 (16.5)
Never	128 (12.4)	689 (66.9)
Quantity		
350 mL	96 (10.6)	13 (3.8)
200 mL	146 (16.2)	40 (11.7)
100 mL	137 (15.2)	99 (29.0)
75 mL	81 (9.0)	153 (44.9)
60 mL	442 (49.0)	36 (10.6)

**Table 3:** Frequency of Saudi coffee consumption by sociodemographic factors (*n* = 1,030).

Sociodemographic data	Frequency of Saudi coffee consumption										
	≥6 times/day (*n* = 38)	4–5 times/day (*n* = 43)	2–3 times/day (*n* = 205)	1 time/day (*n* = 206)	5–6 times/week (*n* = 35)	2–4 times/week (*n* = 160)	1 time/week (*n* = 115)	1–3 times/month (*n* = 100)	Never (*n* = 128)	χ^2^	*p*
	No. (%)	No. (%)	No. (%)	No. (%)	No. (%)	No. (%)	No. (%)	No. (%)	No. (%)		
Gender											
Male	11 (28.9)	15 (34.9)	59 (28.8)	53 (25.7)	14 (40.0)	60 (37.5)	38^a^33.0	25 (25.0)	41 (32.0)	9.99	0.27
Female	27 (71.1)	28 (65.1)	146 (71.2)	153 (74.3)	21 (60.0)	100 (62.5)	77^a^67.0	75 (75.0)	87 (68.0)		
Age (years)											
15–19	2 (5.3)	0 (0.0)	6 (2.9)	5 (2.4)	1 (2.9)	5 (3.1)	5 (4.3)	5 (5.0)	10 (7.8)	97.60	^MC^p > 0.001^*^
20–29	24 (63.2)	24 (55.8)	49 (23.9)	56 (27.2)	12 (34.3)	73 (45.6)	34 (29.6)	47 (47.0)	54 (42.2)		
30–39	5 (13.2)	7 (16.3)	56 (27.3)	43 (20.9)	10 (28.6)	41 (25.6)	35 (30.4)	24 (24.0)	35 (27.3)		
40–49	3 (7.9)	8 (18.6)	44 (21.5)	52 (25.2)	6 (17.1)	23 (14.4)	27 (23.5)	8 (8.0)	13 (10.2)		
50–59	2 (5.3)	3 (7.0)	34 (16.6)	38 (18.4)	6 (17.1)	14 (8.8)	10 (8.7)	10 (10.0)	13 (10.2)		
≥60	2 (5.3)	1 (2.3)	16 (7.8)	12 (5.8)	0 (0.0)	4 (2.5)	4 (3.5)	6 (6.0)	3 (2.3)		
BMI (kg/m^2^)											
Underweight	5 (13.2)	3 (7.0)	4 (2.0)	6 (2.9)	3 (8.6)	5 (3.1)	7 (6.1)	8 (8.0)	18 (14.1)	55.03	>0.001^*^
Normal	21 (55.3)	22 (51.2)	78 (38.0)	73 (35.4)	13 (37.1)	65 (40.6)	39 (33.9)	39 (39.00)	57 (44.5)		
Overweight	5 (13.2)	13 (30.2)	70 (34.1)	73 (35.4)	13 (37.1)	45 (28.1)	40 (34.8)	29 (29.0)	28 (21.9)		
Obese	7 (18.4)	5 (11.6)	53 (25.9)	54 (26.2)	6 (17.1)	45 (28.1)	29 (25.2)	24 (24.0)	25 (19.5)		
Nationality											
Saudi	38 (100)	41 (95.3)	201 (98.0)	202 (98.1)	34 (97.1)	151 (94.4)	106 (92.2)	94 (94.0)	119 (93.0)	14.40	^MC^p = 0.05
Non-Saudi	0 (0.0)	2 (4.7)	4 (2.0)	4 (1.9)	1 (2.9)	9 (5.6)	9 (7.8)	6 (6.0)	9 (7.0)		
Marital status											
Unmarried (Single, divorced, or widowed)	28 (73.7)	23 (53.5)	75 (36.6)	83 (40.3)	17 (48.6)	78 (48.8)	50 (43.5)	61 (61.0)	84 (65.6)	49.74	>0.001^*^
Married	10 (26.3)	20 (46.5)	130 (63.4)	123 (59.7)	18 (51.4)	82 (51.3)	65 (56.5)	39 (39.0)	44 (34.4)		
Educational level											
Lower than high school	1 (2.6)	0 (0.0)	8 (3.9)	10 (4.9)	1 (2.9)	2 (1.3)	6 (5.2)	2 (2.0)	5 (3.9)		
High school	8 (21.1)	9 (20.9)	31 (15.1)	29 (14.1)	3 (8.6)	24 (15.0)	18 (15.7)	13 (13.0)	16 (12.5)	26.61	0.32
Bachelor’s degree	20 (52.6)	28 (65.1)	127 (62.0)	128 (62.1)	26 (74.3)	100 (62.5)	54 (47.0)	64 (64.0)	83 (64.8)		
Higher than a bachelor’s degree	9 (23.7)	6 (14.0)	39 (19.0)	39 (18.9)	5 (14.30)	34 (21.3)	37 (32.2)	21 (21.0)	24 (18.8)		
Work status											
Student	16 (42.1)	8 (18.6)	21 (10.2)	35 (17.0)	7 (20.0)	35 (21.9)	21 (18.3)	24 (24.0)	30 (23.4)		
Working (employee + self-employed)	13 (34.2)	23 (53.5)	118 (57.6)	111 (53.9)	17 (48.6)	80 (50.0)	65 (56.5)	49 (49.0)	62 (48.4)	29.36	0.02^*^
Not working (not working + retired + housewife)	9 (23.7)	12 (27.9)	66 (32.2)	60 (29.1)	11 (31.4)	45 (28.1)	29 (25.2)	27 (27.0)	36 (28.1)		
Monthly income (SAR)											
<2,000	15 (39.5)	13 (30.2)	38 (18.5)	54 (26.2)	10 (28.6)	49 (30.6)	31 (27.0)	32 (32.0)	33 (25.8)		
2,000–5,000	9 (23.7)	7 (16.3)	21 (10.2)	26 (12.6)	6 (17.1)	29 (18.1)	19 (16.5)	14 (14.0)	28 (21.9)	57.94	0.003^*^
5,000–7,000	4 (10.5)	6 (14.0)	19 (9.3)	11 (5.3)	3 (8.6)	8 (5.0)	9 (7.8)	6 (6.0)	9 (7.0)		
7,000–10,000	3 (7.9)	10 (23.3)	27 (13.2)	30 (14.6)	3 (8.6)	13 (8.1)	10 (8.7)	18 (18.0)	14 (10.9)		
>10,000	7 (18.4)	7 (16.3)	100 (48.8)	85 (41.3)	13 (37.1)	61 (38.1)	46 (40.0)	30 (30.0)	44 (34.4)		
Region											
Central	23 (60.5)	27 (62.8)	129 (62.9)	148 (71.8)	21 (60.0)	105 (65.6)	70 (60.9)	45 (45.0)	69 (53.9)		
Northern	1 (2.6)	5 (11.6)	8 (3.9)	6 (2.9)	1 (2.9)	4 (2.5)	3 (2.6)	3 (3.00)	5 (3.9)	51.52	^MC^p = 0.007^*^
Southern	2 (5.3)	4 (9.3)	14 (6.8)	6 (2.9)	1 (2.9)	6 (3.8)	3 (2.6)	5 (5.0)	3 (2.3)		
Western	8 (21.1)	6 (14.0)	34 (16.6)	32 (15.5)	9 (25.7)	28 (17.5)	30 (26.1)	29 (29.0)	37 (28.9)		
Eastern	4 (10.5)	1 (2.3)	20 (9.8)	14 (6.8)	3 (8.6)	17 (10.6)	9 (7.8)	18 (18.0)	14 (10.9)		
Health status											
Healthy (no disease)	33 (86.8)	30 (69.8)	148 (72.2)	149 (72.3)	25 (71.4)	121 (75.6)	93 (80.9)	74 (74.0)	90 (70.3)	8.38	0.40
Unhealthy (diseased state)	5 (13.2)	13 (30.2)	57 (27.8)	57 (27.7)	10 (28.6)	39 (24.4)	22 (19.1)	26 (26.00)	38 (29.7)		

*χ2, Chi-square test; MC, Monte Carlo; BMI, Body mass index; SAR, Saudi Arabian Riyal.*

*p, p-value of the comparison of the frequency of consumption of Saudi coffee in relation to sociodemographic factors.*

^*^Statistically significant at *p* ≤ 0.05.

### 3.3 Frequency of Turkish coffee consumption

The pattern of Turkish coffee consumption differed from that of Saudi coffee consumption. Two-thirds of the respondents (66.8%, *n* = 689) reported never consuming this drink, followed by 16.5% who drank coffee 1–3 times per month (*n* = 170). Additionally, the most common serving size of Turkish coffee consumed was 75 mL (44.9%) (Table 2). Regarding the frequency by sociodemographic data (Table 4), significant differences were observed in age, nationality, marital status, educational level, and region according to the frequency of Turkish coffee consumption (*p* < 0.05). The age group 20–29 years reported the highest percentage of more than half (53.8%) for a frequency of consumption of 5–6 times per week, and 40.6% of those who never consumed Turkish coffee belonged to the same group. In terms of marital status, unmarried respondents represented the majority (84.6%) of those consuming Turkish coffee at a frequency of 5–6 times per week. Therefore, almost equal proportions of unmarried and married respondents reported consuming coffee 1–3 times/week or never (50%). Regarding the educational level, the findings demonstrated that bachelor’s degree holders consumed this drink 5–6 times a week and represented 61.5% of this study subgroup. Respondents earning >10,000 SAR and those from the central region showed the lowest frequency of consumption compared to the other groups (37.0 and 65.0%, respectively).

**Table 4:** Frequency of Turkish coffee consumption by sociodemographic factors (*n* = 1,030).

Sociodemographic data	Frequency of Turkish coffee consumption										
	≥6 times/day (*n* = 38)	4–5 times/day (*n* = 43)	2–3 times/day (*n* = 205)	1 time/day (*n* = 206)	5–6 times/week (*n* = 35)	2–4 times/week (*n* = 160)	1 time/week (*n* = 115)	1–3 times/month (*n* = 100)	Never (*n* = 128)	χ^2^	*p*
	No. (%)	No. (%)	No. (%)	No. (%)	No. (%)	No. (%)	No. (%)	No. (%)	No. (%)		
Gender											
Male	0 (0.0)	2 (40.0)	10 (43.5)	8^a^18.6	6 (46.20)	9 (26.5)	14 (26.4)	48 (28.2)	219 (31.8)	7.99	0.33
Female	0 (0.0)	3 (60.0)	13 (56.5)	35^a^81.4	7 (53.8)	25 (73.5)	39 (73.6)	122 (71.8)	470 (68.2)		
Age (years)											
15–19	0 (0.0)	0 (0.0)	0 (0.0)	0 (0.0)	0 (0.0)	1 (2.9)	1 (1.9)	7 (4.1)	30 (4.4)	62.97	^MC^p = 0.001^*^
20–29	0 (0.0)	1 (20.0)	2 (8.7)	9 (20.9)	7 (53.8)	8 (23.5)	10 (18.9)	56 (32.9)	280 (40.6)		
30–39	0 (0.0)	2 (40.0)	7 (30.4)	14 (32.6)	3 (23.1)	6 (17.6)	12 (22.6)	50 (29.4)	162 (23.5)		
40–49	0 (0.0)	1 (20.0)	6 (26.1)	12 (27.9)	2 (15.4)	11 (32.4)	10 (18.9)	31 (18.2)	111 (16.1)		
50–59	0 (0.0)	1 (20.0)	8 (34.8)	5 (11.6)	0 (0.0)	7 (20.6)	14 (26.4)	17 (10.0)	78 (11.3)		
≥60	0 (0.0)	0 (0.0)	0 (0.0)	3 (7.0)	1 (7.7)	1 (2.9)	6 (11.3)	9 (5.3)	28 (4.1)		
BMI (kg/m^2^)											
Underweight	0 (0.0)	0 (0.0)	0 (0.0)	0 (0.0)	3 (23.1)	0 (0.0)	2 (3.8)	12 (7.1)	42 (6.1)	29.39	^MC^p = 0.07
Normal	0 (0.0)	2 (40.0)	11 (47.8)	20 (46.5)	3 (23.1)	13 (38.2)	16 (30.2)	57 (33.5)	285 (41.4)		
Overweight	0 (0.0)	2 (40.0)	4 (17.4)	10 (23.3)	2 (15.4)	12 (35.3)	23 (43.4)	50 (29.4)	213 (30.9)		
Obese	0 (0.0)	1 (20.0)	8 (34.80)	13 (30.2)	5 (38.5)	9 (26.5)	12 (22.6)	51 (30.0)	149 (21.6)		
Nationality											
Saudi	0 (0.0)	4 (80.0)	17 (73.9)	41 (95.3)	10 (76.9)	32 (94.1)	50 (94.3)	164 (96.5)	668 (97.0)	28.83	^MC^p < 0.001^*^
Non-Saudi	0 (0.0)	1 (20.0)	6 (26.1)	2 (4.7)	3 (23.1)	2 (5.9)	3 (5.7)	6 (3.5)	21 (3.0)		
Marital status											
Unmarried (Single, divorced, or widowed)	0 (0.0)	1 (20.0)	7 (30.4)	16 (37.2)	11 (84.6)	14 (41.2)	20 (37.7)	85 (50.0)	345 (50.1)	17.64	0.01^*^
Married	0 (0.0)	4 (80.0)	16 (69.6)	27 (62.8)	2 (15.4)	20 (58.8)	33 (62.3)	85 (50.0)	344 (49.9)		
Educational level											
Lower than high school	0 (0.0)	1 (20.0)	3 (13.0)	1 (2.3)	1 (7.7)	3 (8.8)	3 (5.7)	2 (1.2)	21 (3.0)		
High school	0 (0.0)	0 (0.0)	2 (8.7)	7 (16.3)	2 (15.4)	4 (11.8)	7 (13.2)	27 (15.9)	102 (14.8)	32.72	^MC^p = 0.02^*^
Bachelor’s degree	0 (0.0)	1 (20.0)	10 (43.5)	26 (60.5)	8 (61.5)	20 (58.8)	25 (47.2)	106 (62.4)	434 (63.0)		
Higher than a bachelor’s degree	0 (0.0)	3 (60.0)	8 (34.8)	9 (20.9)	2 (15.4)	7 (20.6)	18 (34.0)	35 (20.6)	132 (19.2)		
Work status											
Student	0 (0.0)	0 (0.0)	1 (4.3)	3 (7.0)	6 (46.2)	7 (20.6)	5 (9.4)	33 (19.40)	142 (20.6)		
Working (employee + self-employed)	0 (0.0)	4 (80.0)	12 (52.2)	22 (51.2)	5 (38.5)	17 (50.0)	31 (58.5)	92 (54.1)	355 (51.5)	21.80	^MC^p = 0.06
Not working (not working + retired + housewife)	0 (0.0)	1 (20.0)	10 (43.5)	18 (41.9)	2 (15.4)	10 (29.4)	17 (32.1)	45 (26.5)	192 (27.9)		
Monthly income (SAR)											
<2,000	0 (0.0)	0 (0.0)	5 (21.7)	12 (27.9)	5 (38.5)	7 (20.6)	11 (20.8)	44 (25.9)	191 (27.7)		
2,000–5,000	0 (0.0)	2 (40.0)	3 (13.0)	5 (11.6)	2 (15.4)	5 (14.7)	11 (20.8)	20 (11.8)	111 (16.1)	19.76	^MC^p = 0.84
5,000–7,000	0 (0.0)	0 (0.0)	4 (17.4)	2 (4.70)	0 (0.0)	3 (8.8)	2 (3.8)	16 (9.4)	48 (7.0)		
7,000–10,000	0 (0.0)	1 (20.0)	4 (17.4)	7 (16.3)	2 (15.4)	4 (11.8)	4 (7.5)	22 (12.9)	84 (12.2)		
>10,000	0 (0.0)	2 (40.0)	7 (30.4)	17 (39.5)	4 (30.8)	15 (44.1)	25 (47.2)	68 (40.0)	255 (37.0)		
Region											
Central	0 (0.0)	3 (60.0)	11 (47.8)	20 (46.5)	4 (30.8)	19 (55.9)	34 (64.2)	98 (57.60)	448 (65.0)	56.12	^MC^p < 0.001^*^
Northern	0 (0.0)	0 (0.0)	1 (4.3)	4 (9.3)	0 (0.0)	4 (11.8)	1 (1.9)	11 (6.5)	15 (2.2)		
Southern	0 (0.0)	0 (0.0)	2 (8.7)	2 (4.7)	0 (0.0)	3 (8.8)	0 (0.0)	2 (1.2)	35 (5.1)		
Western	0 (0.0)	2 (40.0)	7 (30.4)	16 (37.2)	6 (46.2)	6 (17.6)	14 (26.4)	40 (23.5)	122 (17.7)		
Eastern	0 (0.0)	0 (0.0)	2 (8.7)	1 (2.3)	3 (23.1)	2 (5.9)	4 (7.5)	19 (11.2)	69 (10.0)		
Health status											
Healthy (no disease)	0 (0.0)	4 (80.0)	14 (60.9)	33 (76.7)	11 (84.6)	19 (55.9)	37 (69.8)	123 (72.4)	522 (75.8)	10.74	0.15
Unhealthy (diseased state)	0 (0.0)	1 (20.0)	9 (39.1)	10 (23.3)	2 (15.40)	15 (44.1)	16 (30.2)	47 (27.6)	167 (24.2)		

χ^2^, Chi-square test; MC, Monte Carlo; BMI, Body mass index; SAR, Saudi Arabian Riyal.

*p, p-value of the comparison of the frequency of consumption of Turkish coffee in relation to sociodemographic factors.*

*Statistically significant at *p* ≤ 0.05.

### 3.4 Association of the frequency with the quantity of Saudi and Turkish coffee consumption

The association between the frequency and quantity of Saudi and Turkish coffees was significant for both drinks (Table 5). For Saudi coffee, the lowest quantity (60 mL) was the least frequently consumed (1–3 times/month), followed by the highest frequency consumed (≥6 times/day), reflected by 70 and 63.2%, respectively. In contrast, Turkish coffee showed a lower frequency and higher quantity of consumption as indicated by the percentages, with 75 mL most frequently consumed 1 time/week by 52.8%, followed by 100 mL consumed 5–6 times/week by 46.2%.

**Table 5:** Association of the frequency and quantity of Saudi and Turkish coffee consumption (*n* = 1,030).

Quantity	Frequency of Saudi coffee and Turkish coffee consumption								χ^2^	*p*
	≥6 times/day	4–5 times/day	2–3 times/day	1 time/day	5–6 times/week	2–4 times/week	1 time/week	1–3 times/month		
	No. (%)	No. (%)	No. (%)	No. (%)	No. (%)	No. (%)	No. (%)	No. (%)		
Saudi coffee, mL	(*n* = 38)	(*n* = 43)	(*n* = 205)	(*n* = 206)	(*n* = 35)	(*n* = 160)	(*n* = 115)	(*n* = 100)		
350	3 (7.9)	5 (11.6)	39 (19.0)	24 (11.7)	5 (14.3)	12 (7.5)	5 (4.3)	3 (3.0)		
200	2 (5.3)	8 (18.6)	42 (20.5)	43 (20.9)	3 (8.6)	27 (16.9)	13 (11.3)	8 (8.0)		
100	6 (15.80)	8 (18.6)	28 (13.70)	39 (18.9)	8 (22.9)	24 (15.0)	14 (12.2)	10 (10.0)	73.13	>0.001^*^
75	3 (7.9)	2 (4.7)	20 (9.8)	14 (6.8)	4 (11.4)	15 (9.4)	14 (12.2)	9 (9.0)		
60	24 (63.2)	20 (46.5)	76 (37.1)	86 (41.7)	15 (42.9)	82 (51.3)	69 (60.0)	70 (70.0)		
Turkish coffee, mL	(*n* = 0)	(*n* = 5)	(*n* = 23)	(*n* = 43)	(*n* = 13)	(*n* = 34)	(*n* = 53)	(*n* = 170)		
350	0 (0.0)	2 (40.0)	5 (21.7)	1 (2.3)	2 (15.4)	1 (2.9)	1 (1.9)	1 (0.6)		
200	0 (0.0)	1 (20.0)	5 (21.7)	11 (25.6)	1 (7.7)	5 (14.7)	4 (7.5)	13 (7.6)		
100	0 (0.0)	1 (20.0)	6 (26.1)	11 (25.6)	6 (46.20)	9 (26.5)	15 (28.3)	51 (30.0)	50.67	^MC^p > 0.001^*^
75	0 (0.0)	1 (20.0)	6 (26.1)	17 (39.5)	4 (30.8)	13 (38.2)	28 (52.8)	84 (49.4)		
60	0 (0.0)	0 (0.0)	1 (4.3)	3 (7.0)	0 (0.0)	6 (17.6)	5 (9.40)	21 (12.4)		

χ^2^, Chi-square test; MC, Monte Carlo.

*p, p-value of the comparison of the frequency and quantity of Saudi and Turkish coffee consumption.*

^*^Statistically significant at *p* ≤ 0.05.

### 3.5 Caffeine exposure per kilogram body weight per day

The relationships between caffeine exposure per body weight per day (mg/kg/day) and sociodemographic characteristics are shown in Table 6. The highest exposure was 0.95 mg/kg/d of caffeine among respondents with normal weight, followed equally by 0.89 mg/kg/d, by those with monthly income <2,000 SAR and the female group. In contrast, the lowest exposure was observed in males (0.66 mg/kg/d). The highest exposure to caffeine from Turkish coffee was among non-Saudis (1.31 mg/kg/d; followed by those with less than high school education 1.15 mg/kg/d) and was lowest in the eastern region (0.49 mg/kg/d). In general, all significant *p*-values (<0.05) revealed that caffeine exposure from Saudi coffee was higher than that from Turkish coffee. Significant differences among the mean values were obtained in the gender and BMI categories for Saudi coffee and BMI categories only for Turkish coffee. When the sum of exposure from both Turkish and Saudi coffees was calculated, the maximum exposure was obtained among non-Saudis (2.07 mg/kg/d), followed by 1.87 mg/kg/d among respondents with the lowest educational level. The lowest caffeine exposure was observed in the eastern region (1.17 mg/kg/d).

**Table 6:** Association of caffeine exposure/body weight/day from Saudi and Turkish coffees with sociodemographic characteristics.

Sociodemographic characteristics	No.	Caffeine exposure/weight/day (mg/kg/d)		Z	*p*_2_	Saudi + Turkish coffee
		Saudi coffee	Turkish coffee			
		Mean ± SD	Mean ± SD			Mean ± SD
Gender						
Male	316	0.66 ± 0.63	0.56 ± 1.06	2.345	0.02^*^	1.22 ± 1.20
Female	714	0.89 ± 0.80	0.69 ± 1.21	4.955	<0.001^*^	1.58 ± 1.48
U (*p*_1_)		83423.0 (<0.001^*^)	106640.0 (0.09)			92102.0 (<0.001^*^)
Age (years)						
15–19	39	0.79 ± 0.84	0.54 ± 1.25	1.607	0.11	1.34 ± 1.43
20–29	373	0.87 ± 0.86	0.55 ± 1.13	5.348	<0.001^*^	1.42 ± 1.41
30–39	256	0.81 ± 0.74	0.70 ± 1.14	1.806	0.07	1.51 ± 1.40
40–49	184	0.76 ± 0.64	0.74 ± 1.16	1.060	0.29	1.50 ± 1.39
50–59	130	0.84 ± 0.73	0.74 ± 1.34	1.427	0.15	1.58 ± 1.56
≥60	48	0.67 ± 0.51	0.64 ± 1.07	0.349	0.73	1.31 ± 1.15
H (*p*_1_)		4.45 (0.49)	12.23 (0.03^*^)			2.80 (0.73)
BMI (kg/m^2^)						
Underweight	59	0.84 ± 1.01	0.73 ± 1.35	0.457	0.65	1.57 ± 1.65
Normal	407	0.95 ± 0.86	0.70 ± 1.32	4.682	<0.001^*^	1.65 ± 1.59
Overweight	316	0.74 ± 0.63	0.60 ± 1.05	2.469	0.01^*^	1.33 ± 1.25
Obese	248	0.72 ± 0.64	0.61 ± 0.97	1.959	0.05	1.33 ± 1.20
H (*p*_1_)		42.59 (<0.001^*^)	0.65 (0.89)			13.50 (0.004^*^)
Nationality						
Saudi	986	0.82 ± 0.76	0.62 ± 1.12	6.051	<0.001^*^	1.44 ± 1.37
Non-Saudi	44	0.76 ± 0.84	1.31 ± 1.82	1.954	0.05	2.07 ± 2.14
U (*p*_1_)		19755.5 (0.32)	16716.5 (0.002^*^)			18653.5 (0.12)
Marital status						
Unmarried	499	0.83 ± 0.84	0.67 ± 1.26	3.231	0.001^*^	1.50 ± 1.53
Married	531	0.81 ± 0.69	0.62 ± 1.07	4.433	<0.001^*^	1.44 ± 1.30
U (*p*_1_)		132385.5 (0.98)	130775.0 (0.67)			130859.5 (0.73)
Educational level						
Lower than high school	35	0.72 ± 0.72	1.15 ± 2.06	0.216	0.83	1.87 ± 2.36
High school	151	0.87 ± 0.83	0.55 ± 1.01	2.837	0.005^*^	1.43 ± 1.34
Bachelor’s degree	630	0.83 ± 0.79	0.62 ± 1.16	4.933	<0.001^*^	1.45 ± 1.42
Higher than a bachelor’s degree	214	0.77 ± 0.64	0.71 ± 1.09	1.327	0.18	1.48 ± 1.24
H (*p*_1_)		1.56 (0.67)	4.25 (0.234)			1.48 (0.69)
Work status						
Student	197	0.88 ± 0.88	0.59 ± 1.12	3.274	0.001^*^	1.47 ± 1.39
Working	538	0.82 ± 0.74	0.67 ± 1.19	3.848	<0.001^*^	1.49 ± 1.41
Not working	295	0.77 ± 0.71	0.65 ± 1.16	2.344	0.02^*^	1.43 ± 1.44
H (*p*_1_)		2.23 (0.33)	1.00 (0.61)			0.60 (0.74)
Monthly income (SAR)						
<2,000	275	0.89 ± 0.87	0.65 ± 1.25	3.495	<0.001^*^	1.55 ± 1.57
2,000–5,000	159	0.80 ± 0.76	0.63 ± 1.23	2.216	0.03^*^	1.43 ± 1.42
5,000–7,000	75	0.74 ± 0.64	0.69 ± 1.13	1.051	0.29	1.42 ± 1.24
7,000–10,000	128	0.85 ± 0.77	0.64 ± 1.07	1.851	0.06	1.48 ± 1.32
>10,000	393	0.78 ± 0.70	0.65 ± 1.12	2.946	0.003^*^	1.43 ± 1.36
H (*p*_1_)		3.98 (0.41)	1.00 (0.91)			1.03 (0.91)
Region						
Central	637	0.88 ± 0.82	0.55 ± 1.03	6.864	<0.001^*^	1.43 ± 1.31
Northern	36	0.72 ± 0.72	1.0 ± 1.0	1.546	0.12	1.72 ± 1.32
Southern	44	0.81 ± 0.73	0.57 ± 1.22	1.937	0.05	1.38 ± 1.52
Western	213	0.74 ± 0.67	0.97 ± 1.58	1.114	0.27	1.71 ± 1.79
Eastern	100	0.68 ± 0.55	0.49 ± 0.81	1.680	0.09	1.17 ± 1.0
H (*p*_1_)		3.91 (0.42)	26.26 (<0.001^*^)			8.59 (0.07)

SD, Standard deviation; U, Mann–Whitney U test; H, Kruskal-Wallis test; Z, Wilcoxon signed-rank test; BMI, Body mass index; SAR, Saudi Arabian Riyal.

*p*_1_, *p*-value of the relationship between caffeine exposure/body weight (mg/day/kg) and sociodemographic data.

*p2, p-value of the comparison between Saudi and Turkish coffee consumption.*

*Statistically significant at *p* < 0.05.

Table 7 shows the results of the contributions to the recommended caffeine limit (400 mg/day). The mean value for Saudi coffee ranged between a maximum of 16.26% among those who were obese and a minimum of 9.85% among the underweight. The highest mean value obtained for Turkish coffee was 24.43% among non-Saudi versus the lowest average of 8.38% in the group aged 15–19 years. Generally, all significant *p*-values (<0.05) were obtained, as the mean contribution from Saudi coffee was higher than that from Turkish coffee, except for the non-Saudi group (12.79 vs. 24.43, *p* = 0.02). The comparison of mean values was significant for marital status, work status, and BMI categories for Saudi coffee, whereas for Turkish coffee, significant differences were obtained for nationality, region, age groups, and BMI categories. Respondents belonging to the lowest educational level made the highest contribution (34.82%), followed by the obese group (30.2%), in contrast to the underweight group which had the lowest mean consumption (19.39%).

**Table 7:** Relationship between the percent contribution (%) to the recommended caffeine limit (400 mg/day) from Saudi and Turkish coffees and the sociodemographic characteristics.

Sociodemographic characteristics	No.	% Contribution to recommended caffeine limit (400 mg/day)		Z	*p*_2_	Saudi + Turkish coffee
		Saudi coffee	Turkish coffee			
		Mean ± SD.	Mean ± SD.			Mean ± SD.
Gender						
Male	316	12.98 ± 11.79	11.51 ± 21.14	2.162	0.03^*^	24.49 ± 23.62
Female	714	14.56 ± 13.13	11.27 ± 19.42	4.883	<0.001^*^	25.87 ± 24.37
U (*p*_1_)		104848.0 (0.06)	110568.0 (0.54)			107524.5 (0.22)
Age (years)						
15–19	39	11.50 ± 11.86	8.38 ± 20.51	1.814	0.07	19.89 ± 22.83
20–29	373	13.55 ± 12.88	8.63 ± 17.56	5.386	<0.001^*^	22.25 ± 21.70
30–39	256	14.80 ± 13.07	12.55 ± 20.24	2.172	0.03^*^	27.35 ± 24.63
40–49	184	13.75 ± 11.42	13.86 ± 21.97	0.724	0.47	27.61 ± 26.05
50–59	130	15.90 ± 14.31	13.78 ± 22.13	1.226	0.22	29.68 ± 27.26
≥60	48	12.79 ± 10.54	12.07 ± 19.34	0.032	0.98	24.87 ± 21.08
H (*p*_1_)		8.948 (0.11)	18.730 (0.002^*^)			19.639 (0.001^*^)
BMI (kg/m^2^)						
Underweight	59	9.85 ± 11.67	9.14 ± 19.04	0.393	0.70	19.39 ± 22.40
Normal	407	13.91 ± 13.91	10.32 ± 19.40	4.668	<0.001^*^	24.23 ± 23.39
Overweight	316	13.38 ± 11.48	11.03 ± 19.67	2.392	0.02^*^	24.41 ± 23.26
Obese	248	16.26 ± 14.21	13.94 ± 21.26	1.942	0.05	30.20 ± 26.18
H (*p*_1_)		14.662 (0.002^*^)	8.192 (0.04^*^)			17.600 (0.001^*^)
Nationality						
Saudi	986	14.14 ± 12.70	10.76 ± 18.91	5.905	<0.001^*^	24.92 ± 23.06
Non-Saudi	44	12.79 ± 13.78	24.43 ± 33.91	2.348	0.02^*^	37.22 ± 40.17
U (*p*_1_)		19836.5 (0.31)	16641.5 (0.002^*^)			18992.5 (0.16)
Marital status						
Unmarried	499	13.14 ± 12.84	10.81 ± 19.87	3.223	0.001^*^	24.0 ± 24.07
Married	531	14.96 ± 12.61	11.84 ± 20.04	4.182	<0.001^*^	26.80 ± 24.15
U (*p*_1_)		117400.0 (0.001^*^)	127624.0 (0.22)			119479.5 (0.006^*^)
Educational level						
Lower than high school	35	13.88 ± 14.64	21.04 ± 35.99	0.276	0.78	34.92 ± 41.32
High school	151	14.62 ± 12.96	10.32 ± 17.59	2.542	0.01^*^	24.94 ± 22.67
Bachelor’s degree	630	14.11 ± 13.03	10.52 ± 18.89	4.952	<0.001^*^	24.67 ± 23.45
Higher than a bachelor’s degree	214	13.63 ± 11.43	12.90 ± 20.60	1.109	0.27	26.52 ± 23.24
H (*p*_1_)		1.209 (0.75)	5.146 (0.16)			2.535 (0.47)
Work status						
Student	197	13.0 ± 12.39	9.14 ± 18.30	3.342	<0.001^*^	22.26 ± 21.65
Working	538	14.89 ± 13.15	12.0 ± 20.49	3.764	0.001^*^	26.89 ± 24.29
Not working	295	13.32 ± 12.16	11.61 ± 19.98	1.995	0.046^*^	24.93 ± 25.27
H (*p*_1_)		6.564^*^ (0.04^*^)	2.831 (0.24)			7.541^*^ (0.02^*^)
Monthly income (SAR)						
<2,000	275	13.63 ± 12.71	10.67 ± 20.86	3.644	<0.001^*^	24.30 ± 25.86
2,000–5,000	159	13.74 ± 13.53	10.82 ± 20.28	2.009	0.045^*^	24.71 ± 24.45
5,000–7,000	75	13.12 ± 11.56	11.74 ± 17.89	0.975	0.33	24.86 ± 20.05
7,000–10,000	128	15.0 ± 13.39	11.19 ± 18.74	1.936	0.05	26.19 ± 22.88
>10,000	393	14.41 ± 12.47	11.99 ± 20.0	2.757	0.006^*^	26.41 ± 23.95
H (*p*_1_)		3.623 (0.46)	2.361 (0.67)			5.201 (0.27)
Region						
Central	637	15.15 ± 13.60	9.64 ± 17.85	6.839	<0.001^*^	24.82 ± 22.43
Northern	36	11.17 ± 9.69	15.69 ± 15.18	1.553	0.12	26.86 ± 18.76
Southern	44	13.22 ± 11.46	10.67 ± 22.81	1.957	0.05	23.89 ± 28.45
Western	213	12.54 ± 11.60	17.01 ± 26.22	1.496	0.14	29.55 ± 30.08
Eastern	100	11.98 ± 10.05	8.87 ± 14.53	1.808	0.07	20.85 ± 18.67
H (*p*_1_)		7.098 (0.13)	25.084 (<0.001^*^)			7.689 (0.10)

SD, Standard deviation; U, Mann–Whitney U test; H, Kruskal-Wallis test; Z, Wilcoxon signed-rank test; BMI, Body mass index; SAR, Saudi Arabian Riyal.

*p*_1_, *p*-value of the relationship between the percent contribution to the recommended caffeine limit (400 mg/day) and sociodemographic factors.

*p2: p-value of the comparison between Saudi and Turkish coffees.*

^*^Statistically significant at *p* ≤ 0.05.

## 4 Discussion

This study aimed to assess the frequency and quantity of consumption of two types of coffee, Saudi and Turkish, in Saudi Arabia. Detailed patterns of beverage intake and exposure to caffeine were estimated, and caffeine quantification was performed based on traditional methods of preparation and by using referenced and standard techniques. This study contributes to the understanding of the growing interest in coffee consumption among the Saudi Arabian population (14) while providing an objective assessment of the extent to which Saudi and/or Turkish coffee could increase consumers’ risk exposure to caffeine intake. Despite several previously published papers with a similar focus, limited information is available regarding the association of coffee consumption with sociodemographic parameters (23–25, 32).

Our results clearly demonstrated that Saudi coffee is more frequently consumed than Turkish coffee in the study population. This is evident from both the proportion of respondents consuming Saudi coffee (88%) versus Turkish coffee (33.2%) and the frequency of consumption. The highest proportion was reported consuming Saudi coffee either 5–6 times per week or 1 time per day (40%), while 16% reported consuming Turkish coffee 1–3 times per month. This finding is consistent with the extant literature where Saudi/Arabic coffee known as “Gahwa” embodies hospitality, tradition, and social interaction among Saudis (33) (representing 95.7% of our study population). In addition, it serves heavily at all types of family and social gatherings (34). Regarding consumption patterns, our results indicate the existence of fluctuations across different subgroups of sociodemographic characteristics. The three parameters significantly associated with the consumption patterns of both Saudi and Turkish coffee were marital status, age, and region. However, the two drinks exhibited different patterns. Taken together, these findings suggest the existence of predictive confounders that affect Saudi and Turkish coffee consumption behaviours among these consumers, as reported in other nations (35). Samoggia and Riedel (36) provided a model of the key determinants of coffee consumption, including sociodemographics, personal preferences, economic attributes, product attributes, and the context of consumption (36).

No doubt, given the large and growing number of different sources of caffeine for consumers in Saudi Arabia, an assessment of the level of caffeine exposure from Saudi coffee and Turkish coffee is important, given the frequent and common consumption among the population. This could be perceived as the first step toward understanding the safety of such exposure. The Food and Drug Administration has included caffeine in the list of safe molecules; a precision of 400 mg/day was not generally associated with significant adverse health effects, such as increased risk of cardiovascular diseases, diabetes, or gastrointestinal problems (21, 37). Similarly, the Netherlands Nutrition Center set the recommended safe consumption in milligrams per kilogram of body weight/day at 5.7 for adults and 2.5 for adolescents (38). Many studies have confirmed the positive effects of caffeine intake on humans, including increased alert status, learning skills, workout performance, and enhanced mood. In contrast, when consumed at high doses, caffeine may adversely affect glucose tolerance and increase the elimination of minerals such as calcium via urinary excretion (39–42). Laboratory analyses of our samples revealed an average amount of 49.8 mg caffeine/100 mL in Saudi coffee, which was lower than that in Turkish coffee (129.6 mg/100 mL). Similarly, Naser et al. (43) reported that Saudi coffee contains a very low dose of caffeine compared with Turkish coffee. However, our study demonstrated the existence of a wide spectrum of caffeine exposure that varied significantly across certain sociodemographic characteristics. This variation differed between the two coffees. Interestingly, daily caffeine exposure (mg/kg/day) was significantly higher for Saudi coffee than for Turkish coffee. This could be explained by the high frequency and amount consumed, as the frequency of consumption with the highest number of respondents was ≥6 times/day (63.2%) for Saudi coffee and once per week (52.8%) for Turkish coffee. In general, consuming both coffees conferred no risk of caffeine exposure (range 0.95–0.66 and 1.31–0.49 mg/kg/d for Saudi coffee and Turkish coffee, respectively). Additionally, even when summing up the exposure from both Turkish and Saudi coffees, the maximum exposure obtained equalled 2.07 mg/kg/d, which was less than 2-fold the recommended maximum exposure at 5.7 mg/kg/d (38). Further analysis revealed that the percentage of contribution to 400 mg caffeine per day was low for both coffees (16.26–9.85% for Saudi coffee and 24.43–8.38% for Turkish coffee).

A few limitations of this study should be considered when interpreting the findings. First, because the sample was not chosen through random selection, the key disadvantage of convenience sampling is that the sample lacks clear generalisability and cannot represent the population being studied (the Saudi Arabian population). Second, the anthropometric measurement data were self-reported, which could have led to under- or overestimation of BMI, thus affecting the categorisation of respondents accordingly. This study has several strengths. The sample size of 1,030 respondents including both women and men of different age groups and socioeconomic backgrounds, in addition to covering all regions of Saudi Arabia, was large enough to enable a comprehensive description of dietary patterns related to Saudi and Turkish coffee consumption. In addition, the caffeine content was accurately measured in the prepared coffee samples. Furthermore, to the best of our knowledge, this study is a novel contribution to the literature by comparing the association between the consumption of these two coffees and various sociodemographic variables.

In conclusion, this is the first large population-based study in Saudi Arabia to elucidate Saudi and Turkish coffee consumption with an accurate estimation of caffeine intake from these two beverages. Overall, patterns consumption of these two coffees showed a large variety across sociodemographic characteristics, where Saudi coffee was generally more consumed. Caffeine intake remained below the limit of 400 mg daily even when the values for the consumption of both coffees were combined. Accuracy estimation and exposure were obtained from detailed patterns in terms of quantity and frequency of consumption. Accordingly, the results of this study may contribute to the understanding of the influence of culture and public policies on population consumption tendencies and trends. Saudi Arabia is witnessing a remarkable increase in coffee imports and the adoption of coffee consumption promotion initiatives, inciting further consumption of coffee at the national level. However, because caffeine intake was not calculated for additional existing caffeinated beverages, this aspect should be considered in future studies in addition to incorporating further analysis to predict coffee and caffeine consumption based on sociodemographic characteristics.

The present study is one of the first of its kind, including the 5 regions of Saudi Arabia with a large number of participants. Laboratory analyses were performed to estimate the caffeine content in these two types of coffee to support and complete the intake data. This helped obtain a quantitative estimation of caffeine intake among the studied population. Insight analysis from this study serves as a basis for expanding and developing nutritional education interventions at the community level to rationalise coffee consumption as part of an overall healthy lifestyle, which is foreseen as constituting a primary target in Saudi Vision 2030.

## Data Availability

The original contributions presented in the study are included in the article/supplementary material, further inquiries can be directed to the corresponding author.
